# A qualitative study of women's experiences of living with COPD

**DOI:** 10.1002/nop2.86

**Published:** 2017-05-16

**Authors:** Simen A. Steindal, Jane Österlind, Kristin Halvorsen, Therese Schjelderup, Ellen Kive, Liv Wergeland Sørbye, Alfhild Dihle

**Affiliations:** ^1^ Lovisenberg Diaconal University College Oslo Norway; ^2^ VID Specialized University Faculty of Health Studies Oslo Norway; ^3^ Ersta Sköndal University College Stockholm Sweden; ^4^ Oslo and Akershus University College of Applied Sciences Department of Nursing and Health Promotion Kjeller Norway; ^5^ Diakonhjemmet Hospital Outpatient Clinic Oslo Norway

**Keywords:** COPD, female, home‐care, nursing, patient's experience, qualitative research

## Abstract

**Aim:**

To explore women's experiences of living with chronic obstructive pulmonary disease (COPD) at home.

**Design:**

An explorative and descriptive qualitative design.

**Methods:**

A consecutive sample of nine women with COPD living at home. Data were collected in 2014 using semi‐structured interviews and analysed using a qualitative content analysis.

**Results:**

Three main themes were identified: having a good life with COPD despite limitations; predictability and confidence in getting help; and the struggle to achieve a balance between insight and compliance with management of COPD. These women experienced limitations related to the traditional female role and felt unable to fulfil their own expectations. They experienced a good life despite limitations arising from adaptation and coping strategies. To feel safe, they needed to feel confident that they would receive the necessary help in case of exacerbation of their disease. To enhance compliance with COPD management, the women wanted education that provided specific suggestions.

## 1 INTRODUCTION

Previously, chronic obstructive pulmonary disease (COPD) was regarded typically as a disease of men, but the prevalence is increasing among women (Raghavan & Jain, 2015). Increased tobacco smoking among women and the greater susceptibility of women to pulmonary damage from tobacco smoke and air pollution are the main reasons for such development (Van Haren‐Willems & Heijdra, 2010). People suffering from COPD often experience burdensome symptoms in their daily life, such as breathlessness, fear, depression and pain. These burdens often lead to deterioration of quality of life and functional status (Ek, Sahlberg‐Blom, Andershed, & Ternestedt, 2011; Peruzza et al., 2003). Furthermore, people with COPD could experience increased dependency on others, when the disease worsens (Fraser, Kee, & Minick, 2006). Due to cultural, emotional and biological differences, women may experience COPD, the burdens related to the disease and the decreasing quality of life different from men (American Lung Association, 2013).

### 1.1 Background

Previous studies have described women's experiences of living with COPD by comparing men and women. They suggest that women with COPD experience more severe dyspnoea, higher levels of anxiety and depression, poorer quality of life and more exacerbations than men (De Torres et al., 2005; Di Marco et al., 2006; Naberan, Azpeitia, Cantoni, & Miravitlles, 2012; Raherison et al., 2014). To our knowledge, few studies have explored women's experiences of living with COPD (Jonsdottir & Jonsdottir, 2007; O'Neill, 2002; Sexton & Munro, 1988). A survey of women with COPD showed that loneliness, depression, dyspnoea, fatigue and restricted activity were the women's biggest problems. In addition, their overall health was poor (Sexton & Munro, 1988). Jonsdottir and Jonsdottir (2007) interviewed women with COPD about their experiences of relapsing to a smoking habit. The women reported that COPD controlled their life in a fundamental way as they had to relocate to a smaller home, were dependent on portable oxygen support and felt increased isolation. O'Neill (2002) found that women with COPD enrolled in pulmonary rehabilitation programmes struggled with burdensome symptoms and loss of social activities and relationships. Furthermore, they felt stigmatized by the COPD, tobacco smoking or simply by being female.

A review of the literature indicates that the body of knowledge concerning women's experiences of living with COPD at home is sparse. We were not able to identify studies, which aimed to explore how women with COPD experienced to live at home. Knowledge of what is in particular women's experiences living at home with COPD is crucial. Such knowledge may contribute in care for women in a way that empowers them of coping in their everyday life.

The aim of the study was to explore how women with COPD experience their daily life at home. The research question was: How do women with COPD experience their daily life at home?

## 2 METHODS

### 2.1 Design

This study had a qualitative explorative and descriptive design, with semi‐structured interviews of women with COPD, aiming to provide new knowledge and insights into an area with limited research. Such a design enables researchers to explore and to understand deeply, complex phenomena encountered by patients. Semi‐structured interviews are a relevant data collection method for exploring the experiences of informants and the meaning that they attribute to them (Kvale & Brinkmann, 2012).

### 2.2 Participants and recruitment

The participants were recruited from a general hospital in a large city in Norway. The sampling procedure was consecutive. Two experienced nurses at the hospital recruited the patients. The study was part of a larger study of COPD patients using mixed methods. When informed about the quantitative study, women were informed and invited to join this qualitative study. Women were included if they were able to understand, read and write Norwegian and lived at home. Exclusion criteria were any known cognitive impairment and severe hearing impairment. Nine potential women were approached and all volunteered to take part and were included in the qualitative study. Their mean age was 72 years. A description of the recruited women is summarized in Table 1.

**Table 1:** Description of the women

Participant	Age	Living arrangement	Home‐care services	Safety alarm	Oxygen treatment at home	Social activities
1	69	Alone	Public services Home‐care nursing Administering enteral nutrition	No	No	Rarely outdoors Have to go by taxi to be able to move outdoors
2	70	With partner	Public service Housework one day every third week	Yes	No	Driving car herself when COPD is stable
3	63	Alone	Public service Housework once a week	Yes	No	Rarely outdoors Have to go by wheelchair‐accessible taxi to move outdoors
4	87	Alone	No services	No	No	Driving car herself Socializing with friends Several social activities
5	68	With husband	Private services Housework every second week	No	Yes	Rarely outdoors
6	70	With husband	No	No	No	Socializing with friends Several social activities
7	70	With husband	Home‐care nursing Administering drugs Private service Housework	Yes	Yes	Socializing with neighbours and friends
8	79	With husband	Private service Housework every second week	No	No	Socializing and working with others with COPD Dependant on travelling by car, husband drives her
9	70	Alone	Private service Housework every third week	Yes	Yes	Rarely outdoors Have to go by taxi to be able to move outdoors

### 2.3 Data collection

Data collection was conducted by the first author and took place between January ‐ June, 2014. Each interview lasted from 20 to 90 min and the women decided where to be interviewed. Seven women were interviewed at home, one by telephone and another in the first author's office. Open‐ended questions were used in a semi‐structured interview. The interview guide covered the following themes: health condition and breathing, how to cope with COPD, feeling safe at home with COPD and experiences of healthcare providers at home. It also covered the topics of why it is necessary to be hospitalized with COPD, experiences from the last hospitalization, experiences of the transfer from hospital to home and the first days at home after discharge. During the interview, participants were encouraged to speak freely about their experiences and were given opportunity to reflect on each theme. When necessary, the interviewer asked additional questions for the purpose of clarification. The interviews were audiotaped and transcribed verbatim by the first author. All transcripts have been de‐identified.

### 2.4 Data analysis

The interviews were analysed using qualitative content analysis, using the interpretative contexts identified by Kvale and Brinkmann (2012). The analysis was done in three interpretative contexts: self‐understanding, common‐sense understanding and theoretical understanding.

In the context of self‐understanding, two of the authors (SAS and AD) read the text several times in attempting to capture the individual perspectives of the participants and to get an understanding of the content of the material as a whole. Key words and paragraphs were marked in the text to shorten the transcription, to narrow down the scope and text and to omit less relevant information. This process was guided by the aim of the study and meaningful units were identified. The meaningful units were condensed into descriptions close to the participants' own perception of their statements.

In the common‐sense context, the two authors independently re‐read all interviews focusing on the condensed meaning units, after which categories were assigned using descriptive terminology; for example: relationship and knowing my story. To interpret the meaning of these findings, questions were asked such as: What does this tell us about relationships and the significant meaning of relationships for women with COPD? During this interpretation, patterns emerged, which formed the basis of the sub‐themes and main themes. Categories, sub‐themes and main themes were compared and discussed to ensure reasonable interpretations. The findings are presented in a descriptive way to enable the women's voices to come through.

Finally, for the third context (theoretical), the findings were interpreted and discussed in the light of research on COPD and are presented in the Discussion section.

### 2.5 Ethics

The study was approved by the hospital's research committee and the Regional Committee for Medical Research Ethics. All of the nine women were given both oral and written information about the study and they gave their written, voluntary and informed consent prior to participation. The women were informed about their right to withdraw from the study at any time during the research process.

### 2.6 Rigour

Investigator triangulation was used to enhance credibility. There was a continuous dialogue between the first and last author during the analysis process. Tentative categories, sub‐themes and main themes were discussed and assessed as to whether there might be other competing interpretations. Thereafter, two other authors (KH and JÖ) read the interview transcripts and independently reviewed the analysis process. The authors had diverse research and clinical expertise that facilitated different perspectives during the analysis and interpretation of data. To strengthen credibility, examples of how categories, sub‐themes and main themes were abstracted from meaningful units are shown in Table 2.

**Table 2:** Illustration of the analysis process

Meaningful units	Condensed meaningful units	Categories	Sub‐theme	Main theme
I have an (safety) alarm and some close friends. I have a neighbour who is a friend who lives one floor below. I don't feel unsafe. (P9)	Not unsafe thanks to safety alarm, friends and neighbour	Safe at home	Feeling safe at home	Predictability and confidence in getting help
I've been allowed to go directly to the hospital twice. I've telephoned the emergency phone number sometimes and the paramedics they're fantastic, they've taken care of me and transported me to the hospital. It's reassuring to know. (P1)	Reassuring to be allowed to go directly to the hospital and to phone emergency number	Knowing who to get in touch with		
It was reassuring to know that I can phone (name of nurse) if there's something. You can't just phone a switchboard operator and say I want to talk with this or that person. Now, I know who to talk to and I think that's very nice. (P8)	Reassuring to know who to phone if there is something	Relationship	Feeling confident with the healthcare system	
I wish that when you have severe COPD, there was a person all the time who knew your story. (P3)	A person who knows your story	To know my story		
It was an acute hospital admission. Suddenly, I had fever, it was 39.2 °C (>102.2 °F) when I woke up in the morning. I went to the general practitioner. He said you must be hospitalised immediately. (P6)	Acutely hospitalized by general practitioner	Access to general practitioner		
The only thing (that would make it easier to live with COPD at home) is if I get more breathless and so on, I have drugs, but I wish I could go to the hospital for an emergency thing (for observation and assessment), but I cannot. … If I could telephone the hospital to say that I am very ill, but they just answer that you have to telephone the emergency ward. (P4)	When breathless want to go to hospital for observation and assessment, but cannot as have to go through emergency ward	Difficulties getting access to hospital for assessment		

Data collection and interpretation of the data during interviews may be affected by the interviewers' preconceptions. Therefore, to enhance transparency and reflexivity, the interview guide, the data collection and the data analysis were discussed with the other authors, which additionally facilitated discussions of competing interpretation. The validity of the immediate interpretations was assessed in the dialogue between the participants and the researcher, asking questions during the interviews such as: have I understood you right if … or do you mean …? Transferability was enhanced by descriptions of the participants, collection of data and the process of data analysis, in addition to enriched descriptions of the results with relevant quotations.

## 3 FINDINGS

Three main themes were identified from the data analysis: having a good life with COPD despite the limitations; predictability and confidence in getting help; and the struggle to achieve a balance between insight and compliance with management of COPD. The main themes and sub‐themes are elaborated in Table 3.

**Table 3:** Overview of main themes and sub‐themes

Main themes	Sub‐themes
Having a good life with COPD despite limitations	Limitations in own expectations of the traditional female role Coping in a continuum between living and living locked the illness A good life thanks to adaptation strategies
Predictability and confidence in getting help	Feeling safe at home Feeling confident with the healthcare system
The struggle to achieve a balance between insight and compliance with management of COPD	Perceptions of self‐care Having an action plan for episodes of COPD exacerbation

### 3.1 Having a good life with COPD despite limitations

All the women experienced different levels of impaired energy and dyspnoea, which influenced their role as a housewife and concerns about their appearance and ability to socialize.

### 3.2 Limitations in own expectations of the traditional female role

The participants described limitations related to their own expectation of the female role and role as a housewife. Eight of them told that they were not able to do the housework they used to, because of dyspnoea and other burdens related to their COPD. They needed help from their husband, children, daughter in‐laws, private or public services. Three participants expressed concerns about their home not being as clean and tidy as it used to be:

I have a private one (housekeeper) who comes every third week for two hours and makes the bed and washes clothes. I'm no longer able to do that. To put it mildly, that's very frustrating. (P9)

All women experienced in different ways impaired levels of energy to socialize, to invite friends or to be a proper hostess:

I don't have energy for lots of visitors anymore. To begin to serve guests with coffee, I don't even manage to bend down to find coffee cups. He has to do it (husband), but he's unsteady and not so strong, so I have to ask my friends to cover the table and to tidy up afterwards. It's disturbing. (P5)

Two of the three women who used a portable oxygen supply were not willing to use it outdoors because of concerns about their altered appearance. They felt embarrassed about own expectations of their looks:

I haven't seen so many people; it inhibits me to go outside with the oxygen catheter in the nose. It's probably just me. That's how I am. (P7)

The women struggled to accept that they could no longer live up to their own expectations of the traditional female role from the time before their health deteriorated.

### 3.3 Coping in a continuum between living with and living locked in the illness

The women used different coping strategies to manage everyday life with COPD, as they knew that their health conditions and breathing could deteriorate further in the future. One strategy was to live the illness. Living with the illness implied to have a positive outlook focusing on the present situation and avoid thinking about the possible development of the disease and all the potential problems and worries in the future. One informant who lived the illness said the following:

I just know that my breath is declining. I would rather not think about what's going to happen eventually. I don't want to think about it … I don't mope. I don't know what that is. I'm not bored. I don't know if I'm full of energy, but I do things. (P4)

Another strategy was to live locked the illness by having more worries according to everyday life, focusing significantly on how the disease could possibly develop and reflecting on potential future problems. One informant who lived locked the illness said:

My breathing will get even worse, I'm almost not able to (breathe). That's what worries me. (P5)so that I'll nearly suffocate. Sometimes I feel that

However, few of the women used only one strategy. Their strategies could be placed on a continuum between living and living locked the illness. Living or living locked the illness was additionally connected to social roles, as the women who lived alone were housebound and with a limited social network were inclined to live locked the illness. Those who lived with a husband, had a social network and/or were able to leave their home were more inclined to live the illness.

### 3.4 A good life thanks to adaptation strategies

Despite limitations in everyday life, eight women described moments where they experienced having a good life. They told stories about how they socialized with family or friends, conducted meaningful activities or enjoyed a peaceful atmosphere in their homes:

I actually think I used the inhaler once during the whole day (travelled on a road trip with husband and friends). There was no fuss, no stress or anything like that. (P7)

Experiences of good moments were possible thanks to the women's use of adaptation strategies. They adapted to prevent dyspnoea by practising slow and calm movements, planning activities, using cars or taxis when they had to move outdoors and avoiding situations that they knew could cause dyspnoea. Furthermore, assistive devices, such as shower stools, walking frame, wheelchairs and chair lifts, were important for preventing dyspnoea, reducing energy consumption and enabling them to leave their home. All participants were dependent on travelling by car to be able to move outdoors because of their dyspnoea and lack of energy.

### 3.5 Predictability and confidence in getting help

#### 3.5.1 Feeling safe at home

To feel safe at home, all the women wanted to know who to get in touch with, to feel confident that they would receive sufficient help in case of COPD exacerbation. However, previous experiences of getting help shaped future calls for help. As one remarked:

I fell and used my safety alarm. Just as I had twisted myself up onto a chair, they arrived and asked do you need help? I don't need help, but I did three‐quarters of an hour ago, I answered. If there is something wrong with my breathing, I don't use the safety alarm. I telephone the emergency phone number. (P3)

The women who participated had previously experienced that staff at the emergency phone number sent an ambulance with competent healthcare professionals who made them feel safe while they were quickly transported to the hospital. Furthermore, the women reported that living with a husband or having friends next door, or having the ability to travel directly to the hospital, contributed to safety, predictability and confidence in getting sufficient help in case of disease exacerbation:

I knew that I lived only eight minutes from the hospital. My husband could drive me (if I got ill), because I was allowed to go directly to the hospital to be admitted. (P6)

A key feature for the women to feel safe was that they felt that they could trust that healthcare professionals, family or friends would give help when it was needed.

### 3.6 Feeling confident with the healthcare system

Trust and confidence in the healthcare system was of importance for the women. However, the findings elucidated that this was not always the case. The women described tiresome experiences of impersonal interactions with many healthcare professionals who did not know their name or their medical history. They often had to repeat the same information several times. The women wished that they had a contact person at the hospital, a nurse or another healthcare professional, with whom they had a good relationship, who knew their story and could meet their individual needs in a respectful and caring manner:

I'm always well received. They have me in the emergency room within 15 min, but it made me feel very safe that she was someone I was allowed to phone without nagging, without being regarded as a fussy person. (P3)

The women wanted to be able to telephone a contact person for questions about COPD management or for help to assess whether hospitalization was required. One participant said:

My breathing became poorer and poorer. The next day, I telephoned the nurse and asked her: what do I do? (P9)

Accessibility to the hospital was difficult for the interviewed women. If symptoms of COPD exacerbation occurred, it was important to get an appointment with the family doctor who could then hospitalize them. However, poor accessibility out of office hours could cause insecurity among the women:

I understand that everybody can't just go to the hospital. That's why I talked about it with the senior consultant (at the hospital). If I had a contact person, it would help a lot. If they don't hospitalise me, they could say: come here so we can examine you and you can stay for a few hours and then go home, but it hasn't been done yet; usually I've been admitted. (P5)

Travelling to the emergency ward was not an option because of troublesome symptoms such as lack of energy and dyspnoea, the travelling distance and uncertainty regarding how much time they would have to wait before seeing a physician. The emergency ward was regarded as a gatekeeper that prevented them from getting access to the hospital. Instead, they wanted the possibility of going directly to the hospital, not necessarily to be admitted but for observation and assessment of whether hospitalization was needed.

### 3.7 The struggle to achieve a balance between insight and compliance with management of COPD

#### 3.7.1 Perceptions of self‐care

The women perceived that they had sufficient knowledge about COPD. However, they experienced a struggle between knowing that they had to change their way of living and finding motivation for such a change. They needed to stop smoking, to eat and drink sufficiently, to increase their level of physical activity or to conduct respiratory exercises. Living alone contributed to difficulties with compliance, as they tended to forget to eat, or ate less because it was boring to eat alone:

The health‐care professionals at the hospital probably believe that I drink as much water and lemonade at home as in the hospital, but that's not correct. (P2)

Other women who participated reflected concerns related to their altered appearance caused by the use of corticosteroids and the need to use a portable oxygen supply outdoors. They did not want to use oxygen outdoors and were concerned about spots on their skin related to the use of corticosteroids.

Nurses supervised and reminded them about important issues in COPD management as measures to gain weight, the correct use of inhalers and management of dyspnoea. This contributed to increased knowledge and power that enhanced the women's ability to self‐manage their COPD. However, some participants reported that nurses moralized or told them to stop smoking without giving any suggestions on how to quit:

Nurses should give suggestions about how to stop smoking, to minimise and to change habits. They should add something more or say it in a completely different way so you don't feel so looked down on. (P2)

The participants experienced education that provided advice or suggestions about how changing their lifestyle could enhance their ability to comply with COPD management.

### 3.8 Having an action plan for episodes of COPD exacerbation

The women described it as challenging to obtain assessment of the severity of symptoms of COPD exacerbation and whether it was necessary to seek medical help to be hospitalized. They were concerned about being perceived by healthcare professionals as someone who exaggerated their symptoms, nagged or simply wanted to be in hospital rather than at home. The women had received an action plan for COPD exacerbation at the hospital. This was a self‐assessment tool that enabled the women to start treatment at home. This tool increased the women's self‐efficacy by empowering them to trust their own symptom experience, to start treatment at home and to trust themselves when making a decision that it was necessary to ask for medical help. One participant who used her action plan every day said:

This is a self‐management thing that I use and it helps me.… I need to telephone and inform my family doctor or the hospital, but I don't have to go there. I use it almost every day, thinking: how am I today? (P3)

Another felt insecure and did not manage to use her action plan:

When I got ill now, I tried with those medications for a short time. I didn't feel confident when I had to take double doses. I went to the family doctor and he immediately admitted me to the hospital. (P8)

Most women experienced empowerment to comply with COPD management through education, supervision and use of the action plan.

## 4 DISCUSSION

Our findings add knowledge to the literature about women's experiences of restrictions in daily life related to limitations in the traditional female role. These restrictions were related to housework, ability to socialize and concerns about appearance. The women struggled to accept that they were no longer able to live up to their own expectations of this role and tried to live as usual, even though they experienced limitations that prevented them from engaging in meaningful activities. Previous research suggested that people with COPD do not often succeed in maintaining a normal image, which could result in their feeling vulnerable, frustrated and hurt and further isolated (Gysels, Bausewein, & Higginson, 2007).

Doing housework, being a hostess and concerns about appearance could be regarded as feminine qualities. These qualities may in particular be important for women in the generation of our participants. Additionally, this may account for the findings that the women with COPD experienced limitations in their traditional female role. Our assertion is in line with previous studies which found that women with chronic illness experienced sadness and loss regarding their decreased ability to do housework and to adhere to female norms such as nurturing, sensitivity to others and selflessness (Clarke & Bennett, 2013; Roberto & McCann, 2011) . Women with long‐term illness seem to share a concern about their altered appearance, which could limit their ability to achieve and maintain an idealized standard of feminine beauty (Clarke & Bennett, 2013; Clarke & Griffin, 2008). To be aware of such values and concerns should be highlighted when caring for women with COPD.

The women in our study appear to have felt embarrassed by the visibility of their breathing efforts and the need to use a portable oxygen supply outdoors which could have contributed to concerns about their appearance. This could have restricted them to socialize with others as described by Nicolson and Anderson (2003) and Disler et al. (2014). Another explanation could be that the women could have struggled to balance between having control and surrendering into the hands of others regarding housework and being a hostess. Ek et al. (2011) pointed out that need for control could be about making one's own choices according to prior habits and achieving a sense of freedom.

The women's use of coping strategies appears to have contributed to a good life. The women coped in a continuum between living and living locked the illness. Few lived either totally or locked the illness. The women who were inclined to live locked the illness appeared to have been reflecting about the past and lamenting their lost dreams as described by Disler et al. (2014). Inability to maintain previously enjoyed interests and social activities could lead to meaninglessness and loneliness as emphasized by Elofsson and Ohlen (2004). These women could have felt that, despite their efforts to gain control over their illness, they had lost control because of the unpredictable nature of COPD (Fraser et al., 2006). In contrast, those who were inclined to live the illness seemed to remain positive despite their limitations. Our findings thereby elucidate that the women who came to terms with their new female role managed better to cope with their everyday life. Interestingly, these findings correspond to Disler et al. (2014) that coping strategies such as joyful activities may alleviate negative feelings. Additionally, distractions may reduce symptoms of distress (O'Neill, 2002). Those who used strategies related to living the illness could have experienced a sense of connectedness with social relationships and the ability to perform meaningful activities, which could have helped with experiences of happiness and belonging as described by Elofsson and Ohlen (2004).

Consistent with previous research (Barnett, 2005; Ek et al., 2011; Fraser et al., 2006), we found that the women experienced limitations in daily life imposed by their burdensome symptoms. However, we also found that almost all the women in our cohort experienced moments of a good life thanks to adaptation strategies that helped to prevent dyspnoea. Similarly, O'Neill (2002) described women's strategies such as resting, pacing oneself, restricting activities and energy‐conserving modifications to control their symptoms. Adaptation strategies could have helped the women to maintain meaning and hope in their life. Also, people with COPD need to redefine their hopes into realistic and obtainable objectives and to let go of dreams that are no longer possible (Milne, Moyle, & Cooke, 2009)**.**

Our findings underline the importance of knowing who to get in touch with to feel safe and confident in receiving help in episodes of COPD exacerbation when living at home, which is consistent with previous findings (Gruffydd‐Jones, Langley‐Johnson, Dyer, Badlan, & Ward, 2007; O'Neill, 2002). Similar to the results of Ek et al. (2011), the women in our study pointed out the importance of having healthcare professionals knowing their story. The women reported knowing that their health and breathing could deteriorate further. Being aware that each episode of acute dyspnoea could be fatal, together with having to decide whether emergency hospitalization might be necessary, could cause great emotional and psychological stress (Hasson et al., 2008).

The women experienced a struggle in daily life to comply with COPD management. Therefore, they appreciated when nurses reminded them and pointed out important issues about COPD management. Such self‐management education has been described as crucial for changes in behaviour and in improving well‐being (Effing et al., 2012). In contrast to previous qualitative studies (Fraser et al., 2006; Wong et al., 2014), all the women in our study had an action plan for dealing with episodes of COPD exacerbation and only one woman reported that she did not feel confident when her breathing weakened. Discussions between healthcare professionals and patients could increase people's understanding of their condition (Disler et al., 2014). People's knowledge about COPD could increase through education provided by healthcare professionals (Hermiz et al., 2002). Additionally, education could increase patients' feelings of being self‐confident and empowered, which could increase hope for enjoying a normal life (Milne et al., 2009). A prerequisite for self‐management is a partnership between people and healthcare professionals where the healthcare professionals must provide a supporting role (Effing et al., 2012). Health behaviour changes could be a challenging topic to address. Women could be vulnerable because of experiences of self‐blame and of blame from healthcare professionals and from the attitude in society that COPD is self‐inflicted (Halding, Heggdal, & Wahl, 2011; O'Neill, 2002). They might feel stigmatized simply because they are women (O'Neill, 2002). Nurses might assume that increasing knowledge by providing facts and information automatically encourages people with COPD to make changes in health behaviour (Young et al., 2015). However, education perceived as preaching could result in counter‐effects such as women becoming less interested in changing their behaviour (Jonsdottir & Jonsdottir, 2007). Therefore, it is important to explore and understand the difficulties that people experience, which influence their efforts to change their behaviour (Eklund, Nilsson, Hedman, & Lindberg, 2012).

### 4.1 Limitations

Because the women were recruited from an urban area served by a single hospital, it is possible that women from other locations might experience living with COPD differently. However, whether our findings are representative of women with COPD in the wider community depends on the extent to which they are experienced as familiar and interpreted as meaningful by other women with COPD and professionals who meet such women in their work. If these findings are recognized and their usefulness is acknowledged, they could be generalized in the framework of qualitative research (Sandelowski, 1986).

## 5 CONCLUSION

These women with COPD experienced limitations in daily life associated with traditional female role and struggled to accept that they were no longer able to live up to their own expectations in this. They used adaptation strategies and coping strategies which enabled them to experience a good life despite their limitations. Adaptation strategies were in particular actions, which were performed to prevent dyspnoea. Coping strategies were related to how the women perceived their present situation and how they thought about future development of the disease, potential problems and worries. To feel safe in their daily life, the women needed to feel confident that they would receive help in episodes of COPD exacerbation. In addition, they wanted to have access to a contact person who knew their story. To enhance compliance with COPD management, the women wanted education and supervision that provided suggestions and advice instead of being told that they simply had to change their behaviour.

Nurses should be aware that women with COPD in addition to experience problems and concerns because of their illness could struggle to accept that they no longer are able to live up to their own expectations of the traditional female. Women may need particular support from nurses to reconcile and accept the loss of their traditional female role. Education and supervision should take into consideration that altered appearance could influence women's ability to comply with clinical directives. To enhance women's feeling of being safe at home and prevent hospital readmissions, women could profit from having a nurse as a contact person at the hospital, or to be attended to in the emergency room during the evening and night to be assessed for their exacerbation of COPD.

## CONFLICTS OF INTEREST

The authors report no conflicts of interest.

## AUTHOR CONTRIBUTIONS

Design: SAS, LWS and AD; data collection: SAS; analysis: SAS, JÖ, KH and AD; manuscript preparation: SAS, JÖ, KH, TS, EK, LWS and AD.

All the Authors have agreed on the final version and meet at least one of the following criteria [recommended by the ICMJE (http://www.icmje.org/recommendations/)]:

- substantial contribution to conception and design, acquisition of data or analysis and interpretation of data;
- drafting the article or revising it critically for important intellectual content.
